# Lactic Acid Accumulation During Exhaustive Exercise Impairs Release of Neutrophil Extracellular Traps in Mice

**DOI:** 10.3389/fphys.2019.00709

**Published:** 2019-06-12

**Authors:** Yue Shi, Hui Shi, David C. Nieman, Qiongyi Hu, Luyu Yang, Tingting Liu, Xiaofeng Zhu, Hongzhan Wei, Die Wu, Fei Li, Yanqiu Cui, Peijie Chen

**Affiliations:** ^1^ School of Kinesiology, Shanghai University of Sport, Shanghai, China; ^2^ Department of Rheumatology and Immunology, Ruijin Hospital, Shanghai Jiao Tong University School of Medicine, Shanghai, China; ^3^ Human Performance Laboratory, Appalachian State University, North Carolina Research Campus, Kannapolis, NC, United States; ^4^ Department of General Surgery, Huashan Hospital, Cancer Metastasis Institute, Fudan University, Shanghai, China; ^5^ Normal College, Jiaxing University, Jiaxing, China

**Keywords:** lactic acid, neutrophil extracellular traps, exhaustive exercise, ROS, cell-free DNA

## Abstract

Lactic acid (LA) is a sensitive indicator of exercise intensity and duration. A single bout of prolonged and intensive exercise can cause transient immunosuppression through the interaction of cellular, humoral, and hormone factors. Exercise-induced influences on neutrophil extracellular traps (NETs) release have been reported, but the underlying mechanism is unknown. This study investigated NETs release, cell-free DNA (cf-DNA), and LA concentration in mice after 60 and 145 min of intensive, graded treadmill running. The concentration of LA and cf-DNA increased, while the level of myeloperoxidase-DNA (MPO-DNA) (an indicator of NETs release) decreased during 145 min of exhaustive running. LA was positively and negatively correlated with cf-DNA and MPO-DNA (*R*^2^ = 0.57 and 0.53, respectively, both *p* < 0.001). Subsequent *in vitro* experiments were conducted with neutrophils activated by phorbol myristate acetate (PMA) in the presence of LA at different concentrations. Increasing LA concentrations were associated with decreases in NETs release and reactive oxygen species (ROS) formation. Taken together, this work furthers our understanding of how NETs and oxidative reaction respond to one bout of prolonged and intensive running. The data support a negative relationship between LA accumulation and NETs release after heavy exertion.

## Introduction

A single, acute bout of prolonged, strenuous exercise has a temporary depressive effect on immune function including cell-mediated immunity, hormonal-mediated immunity, and hormonal changes (Nieman et al., 1989, 1993; Gleeson, 2007). An acute bout of strenuous physical activity is accompanied by a substantial increase in the number of circulating leukocytes, especially neutrophils. A novel neutrophil immune response termed neutrophil extracellular traps (NETs) was first reported in 2004 (Brinkmann et al., 2004). NETs are released by stimulated neutrophils and include three-dimensional network structures with a DNA skeleton and histone and granular proteins. NETs are capable of entrapping exogenous bacteria, allowing neutrophils to kill while minimizing damage to host cells. The NETs activation and release process (NETosis) begins with nicotinamide adenine dinucleotide phosphate (NADPH) oxidase activation of protein-arginine deiminase 4 (PAD4) *via* reactive-oxygen species (ROS), followed by chromatin decondensation aided by granule protein such as myeloperoxidase (Beck et al., 1999) and neutrophil elastase (NE) (Fuchs et al., 2007; Steinberg and Grinstein, 2007). Eventually, NETs are released through cell membrane rupture and cell death, and cleared away by macrophages, a process lasting for hours depending on the stimulus intensity.

Moderate NETs release is typically beneficial to immune defense, but this process can be deleterious and has been linked to the development of autoimmune diseases and thrombosis (Fuchs et al., 2012; Jorch and Kubes, 2017). Acute and chronic exercise training is a physiological stimulus that increases cell-free DNA (cf-DNA) levels in blood. High intensity running is linked to mechanical muscular damage, leukocyte inflammatory responses, and DNA damage caused by oxidative stress leading to an increase in cf-DNA concentrations. NETs are a part of cf-DNA, but the contribution at different conditions needs clarification. Beither et al. showed an acute and transient increase in cf-DNA and neutrophils displaying morphological signs of NETs release in both endurance-trained and healthy sedentary individuals after 60 min of intensive cycling. Syu et al. also reported that intensive cycling bouts facilitated NETs formation in sedentary but not active subjects. The reason for the inconsistent results in human subjects may be that different NETs detection methods were utilized with large inter-individual variation. Additionally, study methods did not include levels of the MPO-DNA complex, a more direct indicator of NETs production.

To extend scientific understanding in this area, we designed a study to identify the impact of exercise on NETs formation using direct method, with an emphasis on underlying mechanisms including the potential effect of lactic acid. Haller et al. (Haller et al., 2018) reported a correlation between post-exercise increases in cf-DNA and lactate (LA), but offered no biochemical pathway explanation. Animal-based and cell culture experiments have the potential to improve scientific understanding on the linkage between NETs release and exercise and principal mechanisms. Building on prior investigations, this study investigated NETs release, cf-DNA, MPO-DNA, and LA concentrations in mice after 60 and 145 min of intensive, graded treadmill running to a state of exhaustion. Besides, the correlation between LA concentration and NETs formation along with the underlying mechanism were also explored.

## Materials and Methods

### Animals

Fifty male C57BL/6 mice (seven-week-old) were purchased from Shanghai JCJ laboratory animal center and were fed for 1-week acclimatization phase (environmental temperature 20–25°C with a 12 h light/dark cycle and free access to standard pellets and drinking water). The mice were randomly divided into five groups (*n* = 10): (C) Control, E60 (incremental running for 60 min), EE (incremental running until exhaustion, about 145 min), 1.5E (1.5 h recovery after exhaustion), and 3E (3 h recovery after exhaustion). All groups except group C were adapted to 10 min low speed (10–15 m/min) treadmill running for 3 days 1 week at 5° inclination. Animals were maintained and used in compliance with Ethics Committee for Animal Experimentation of Shanghai University of Sport in accordance with the Guide for the Care and Use of Laboratory Animals (Institute for Laboratory Animal Research, USA), and all experimental procedures were approved by the Ethics Committee of Shanghai University of Sport (2018011).

### Exercise Program

**Figure 1:**
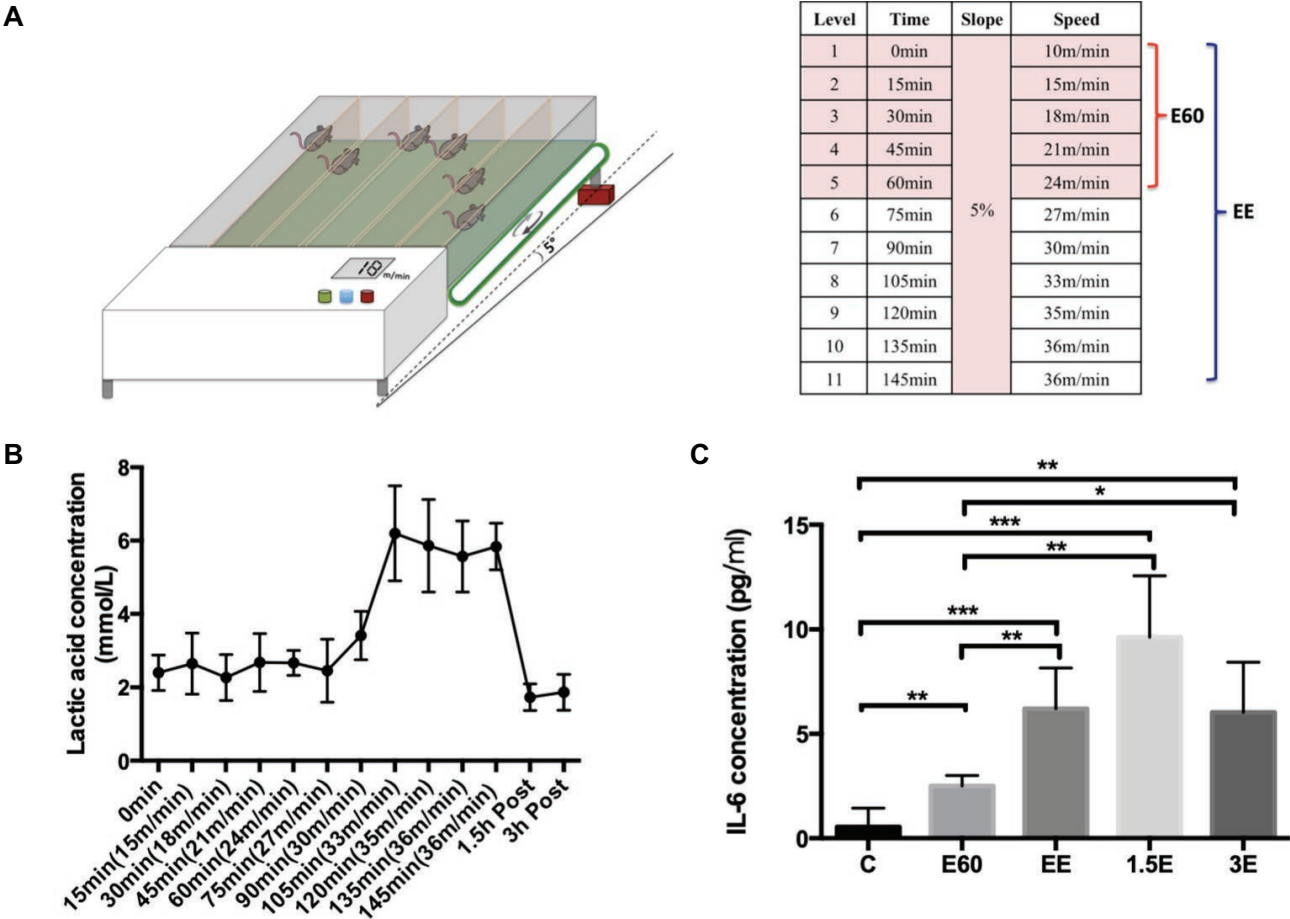
Exhausted treadmill running protocol, LA and IL-6 expression levels. **(A)** The mouse treadmill used in this study was equipped with six separate lanes and the capacity to change the running speed and slope. After a 15-min warmup, the running speed was increased 3 m/min every 15 min with the slope set at 5%. Group E60 finished the first six levels while group EE finished all 11 levels. **(B)** The blood lactic acid concentration was determined by the EFK Lactate Scout using blood drops obtained from the mouse tail after each speed level and after 1.5 and 3 h recovery. Data are expressed as the mean ± SEM. **(C)** Plasma IL-6 concentrations were tested by Quantikine ELISA kit. Data are expressed as the mean ± SEM.

The incremental treadmill running protocol was based on a maximal running capacity test before the formal research. The speed protocol started from 10 m/min to warm-up for 15 min and then changed to 15 m/min running for 15 min. After that, every 15 min, the speed increased 3 m/min, with the slope fixed at 5° inclination. Mice from group E60 finished running for 60 min at the speed of 24 m/min. Mice from group EE ran until exhaustion. When the mouse was nearly exhausted, the speed remained unchanged until exhaustion or the inability to remain on the treadmill belt occurred despite stick stimulation and short rest periods (<1 min) (Lim et al., 2016). Mice from C group were handled and exposed to the static treadmill to control for stress of treadmill environment, while the mice from exercise group were running and anesthetized approximately during the same period. The schematic representation of the exercise program is shown in Figure 1A.

### Detection of Lactic Acid (LA) Concentration

LA concentration was tested immediately after each speed level for 10 s per mouse. LA was detected through test strips using a Lactate Scout+ (EFK Diagnostics, Magdeberg, Germany). Tail venous blood of mice (0.5 μl) was obtained through making a cut with a knife. After wiping away the first drop of blood, a second blood drop was collected with the test strip and LA measured with the Lactate Scout+ in 5 s.

### Serum Preparation and Isolation of Mouse Neutrophils

Immediately after the running bout, mice were removed from the treadmill and anesthetized with 2.5% chloral hydrate (0.1 ml/10 g), with peripheral blood collected from the retro-orbital sinus. For the recovery groups, mice were sacrificed 1.5 and 3 h after the exhaustive running. Serum was isolated from the blood sample and peripheral blood-derived neutrophils were isolated using sterile-filtered sucrose 1.119 and 1.077 g/ml density gradient centrifugation. Bone marrow-derived neutrophils were isolated in accordance with the published methods (Swamydas and Lionakis, 2013), the purity of neutrophils was more than 85% (Supplementary Figure S1).

### Measurement Serum cf-DNA Concentrations

Serum cf-DNA concentrations were measured using the Quant-iT PicoGreen dsDNA assay kit (Life technology). Diluted serum (1:10) was mixed with the PicoGreen dye and added to the microplate wells to make the final volume of 200 μl per well. After the microplate was incubated in the dark for 10 min, the fluorescence of the samples was measured at 480 nm excitation and 520 nm emission using BioTek Synergy2 (USA) (Zhang et al., 2014).

### Serum MPO-DNA Complexes Assay With ELISA Methods

Capture ELISA was used to measure the MPO-DNA complexes as described (Kessenbrock et al., 2009). Briefly, anti-myeloperoxidase antibody (Abcam) was diluted and added into an ELISA plate overnight. After blocking, diluted serum samples were added to the plate overnight. The supernatant was discarded, and diluted PicoGreen with 1 × TE buffer was added to the plate. Fluorescent of the samples was measured by BioTek Synergy2 (USA).

### Analysis of IL-6 Expression

IL-6 expression of mice serum was detected using the Quantikine ELISA kit (R&D, catalog number: M6000B) using instructions provided by the manufacturer. Briefly, 50 μl of the standard, control, serum, and assay diluent mixture were added in the center of each well. After 2 h incubation, the mixture was aspirated, and the wells washed. The mouse IL-6 conjugate was then added to each well and incubated for another 2 h. After aspiration and washing 100 μl of the substrate solution was added. Finally, the stop solution was added with the optical density measured using a microplate reader set to 540 nm.

### Quantification of NETs Release *in vitro*

A cell impermeable DNA binding SytoxGreen dye (Life Technologies) was used for visualizing and measuring NETs release under different conditions. A volume of 100 μl media containing 1 × 10^5^ neutrophils was mixed with 5 μM SytoxGreen and seeded into 96-well plates. These neutrophils were activated with media alone (negative control), 100 nM PMA alone, and 100 nM PMA with LA at different concentrations (5, 10, 15, and 20 mM), respectively. Fluorescence was measured by BioTek Synergy2 (488 nm excitation and 525 nm emission, USA) to assess NETs release. Images were obtained using an Olympus microscope (IX73). The percentage of NETs was calculated as the average of 5 to 10 fields (×400) normalized to the total number of neutrophils, and results were expressed as mean ± SEM.

### Detection of Reactive Oxygen Species Production

DCFH-DA (Beyotime Institute of Biotechnology, Shanghai, China) was used for detecting total reactive oxygen species (ROS) following the manufacturer’s instructions. Briefly, the neutrophils were preloaded with DCFH-DA in serum-free RPMI media. After washing the extracellular DCFH-DA dye, cells were resuspended in fresh RPMI media and 100 μl of 1 × 105 cells were seeded into 96-well plates. These cells were activated with either only media (negative control), PMA or LA for 4 h. The fluorescence was measured by BioTek Synergy2 (USA) to assess the ROS generation.

### Statistical Analysis

All data were analyzed statistically using the SPSS version 20.0 (SPSS Inc., Chicago, IL, US). Quantitative data were expressed as mean ± SEM (standard error of mean). Data with a Gaussian distribution were analyzed by unpaired *t*-test or ANOVA (one-way analysis of variance), while nonparametric data were assessed by Mann-Whitney U test or Wilcoxon rank-sum test. *p* less than 0.05 was considered statistical difference.

## Results and Discussion

LA and IL-6 were used to elucidate the physiological changes during and after the running bout. The LA concentration stayed near 2 mmol/L during the first 75 min of running with the speed below 27 m/min. As the running speed increased from 27 to 33 m/min, the LA concentration increased strongly to about 6 mmol/L and remained near this level until exhaustion. After 1.5 and 3 h recovery from the exhaustive running bout, LA concentrations returned to near normal levels (Figure 1B), the LA concentration of the control group was measured using similar procedures to those with the exercise group, and averaged 2 ± 0.3 mmol/L. The lactate threshold (LT) velocity was defined as the speed at which an increase of >1 mM occurred when LA concentration was between 3.5 and 5 mmol/L. In this paper, LT is regarded as equivalent to the anaerobic threshold (Billat et al., 2005). For purposes of this study, the speed of 27 m/min can be regarded as the LA threshold (LT) velocity. E60 group mice exercise under the LT, with the EE group above the LT. Plasma IL-6 (Figure 1C) increased during and after the running bout, with peak levels measured after 1.5 h recovery and some attenuation after 3 h recovery compared to the C group. The large increases in LA and IL-6 reflected the high physiological demands associated with the exercise bout utilized in this study.

**Figure 2:**
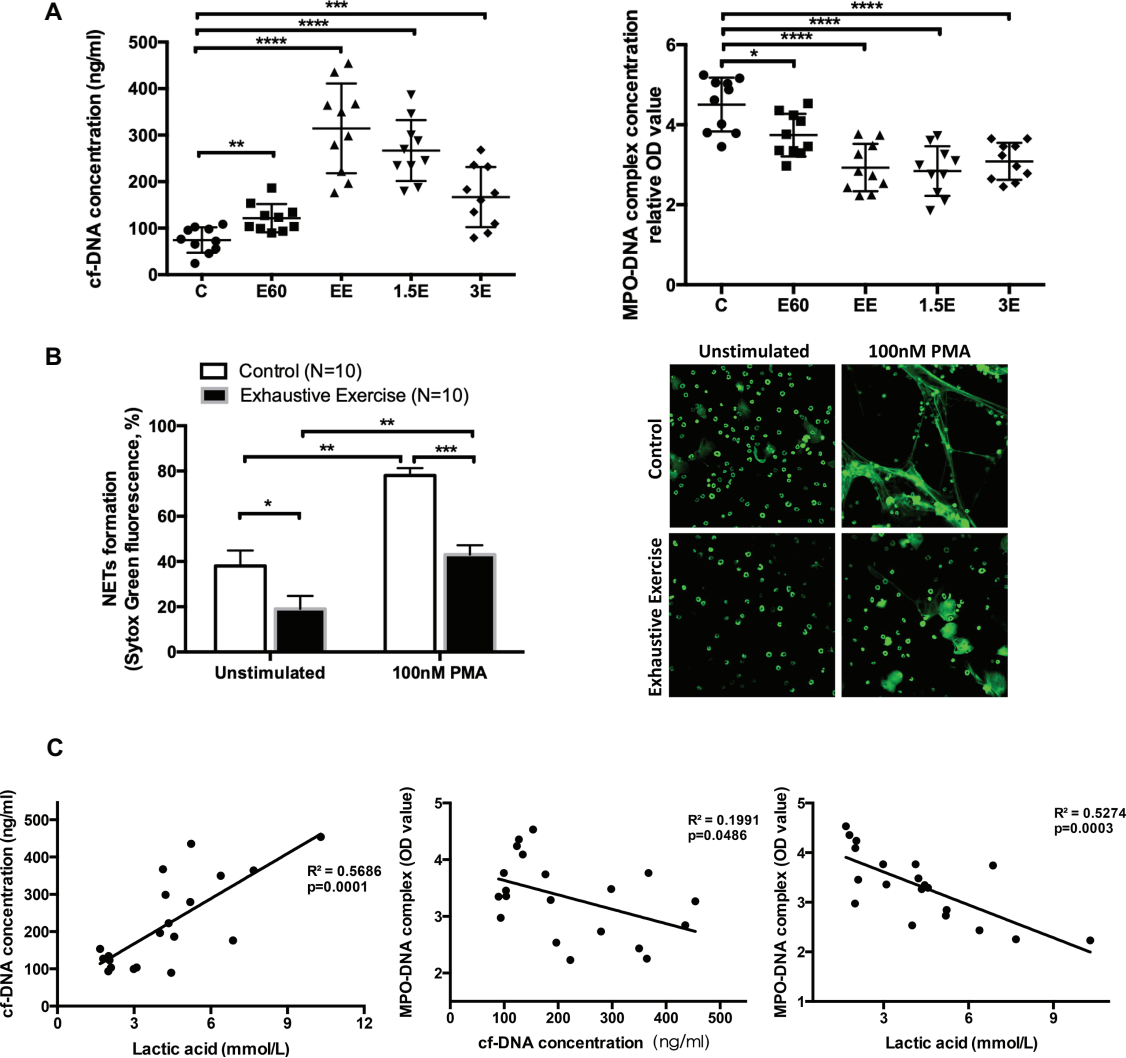
Levels of cell-free DNA (cf-DNA) and MPO-DNA and correlation of cell-free DNA, LA, and MPO-DNA complex values. **(A)** The concentrations of serum cf-DNA were determined by PicoGreen. The serum levels of NETs and MPO-DNA complexes were measured by antibody capture ELISA methods. The symbols represent individual samples, and the data are expressed as mean ± SEM. C, control group; E60, incremental treadmill running for 60 min; EE, exhaustive incremental running for about 145 min; 1.5E, 1.5 h recovery after exhaustive running; 3E, 3 h recovery after exhaustive running. **(B)** NETs release was determined using a fluorescence microplate reader or observed by immunofluorescence microscopy with SytoxGreen extracellular DNA staining. Neutrophils isolated from fresh peripheral blood from the Control **(C)** group or EE group mice were seeded into 96-well plates and treated with media only or 100 nM PMA for 4 h. Original magnification 400×. **(C)** The correlation between concentrations of cell-free DNA, LA, and MPO-DNA complex values. * indicates *p* < 0.05, ** indicates *p* < 0.001, *** indicates *p* < 0.005, and **** indicates *p* < 0.0001.

Serum cf-DNA concentrations increased slightly after 60 min, with the highest levels attained after 145 min of intensive running. After 1.5 and 3 h recovery, cf-DNA levels decreased gradually but were still higher compared to group C (Figure 2A), similar to results previously reported in human athletes (Beiter et al., 2014). In contrast, MPO-DNA decreased after 60 and 145 min of running and remained at low levels 1.5 and 3 h post-exercise (Figure 2A). Quantification of NETs release using the *in vitro* assay (unstimulated with media alone or 100 nM PMA) revealed lower levels in the EE group compared to controls (Figure 2B).

The increase in post-exercise serum cf-DNA is a potential biomarker for overtraining and related immune perturbations (Breitbach et al., 2012). Other contributors to increase in serum cf-DNA include cell death from apoptosis or necrosis processes (Atamaniuk et al., 2004, 2008; Beiter et al., 2011). Thus, the DNA released from NETs with acute exercise may account for only a small but undefined proportion of serum cf-DNA levels.

Correlation analyses were conducted to determine if post-exercise blood LA levels (E60 and EE groups) were related to cf-DNA and MPO-DNA (Figure 2C). LA was positively and negatively correlated with cf-DNA and MPO-DNA (*R* ^2^ = 0.57 and 0.53, respectively, both *p* < 0.001). In consistent with our result, Haller et al. (Haller et al., 2018) reported a high positive correlation between cf-DNA and LA in football players. Besides, recreational runners were also observed similar kinetics for cf-DNA and LA during 10-km cross-country run (Beiter et al., 2011). However, aerobic exercise with stable low concentration of LA also showed a continuous increase of cf-DNA (Haller et al., 2017). Because of the non-standardized exercise mode and detection methods, the accurate origin and reaction of cf-DNA release during exercise still remain to be clarified. NETs, as one of the exact sources of cf-DNA, showed a surprising reverse correlation with LA in our study. The correlation analysis in our work only involves E60 and EE groups, which represents the middle stage and late stage of long duration and high-intensity exercise. LA and NETs value of basal and recovery group did not show obvious correlation as for the rate of NETs returning to normal baseline level in the recovery group was slower than that of blood lactate reduction. Niels Borregaard et al. (Niels Borregaard, 1982) showed that acid environment inhibited the release of NETs through diminishing glycolysis metabolism and ROS production in neutrophils. Accordingly, we speculate that incremental load exercise for more than 2 h consumes a lot of glycogen *in vivo*, both myoglobin and blood sugar concentration will be affected. Therefore, when carbohydrate is not replenished in time after exercise, the requirement of neutrophils for glycogen was not fully met, and glycolysis metabolism was still inhibited to some extent. Therefore, the MPO-DNA level did not return to normal 3 h after exercise in this study, not in sync with the rate at which blood LA drops to normal level.

**Figure 3:**
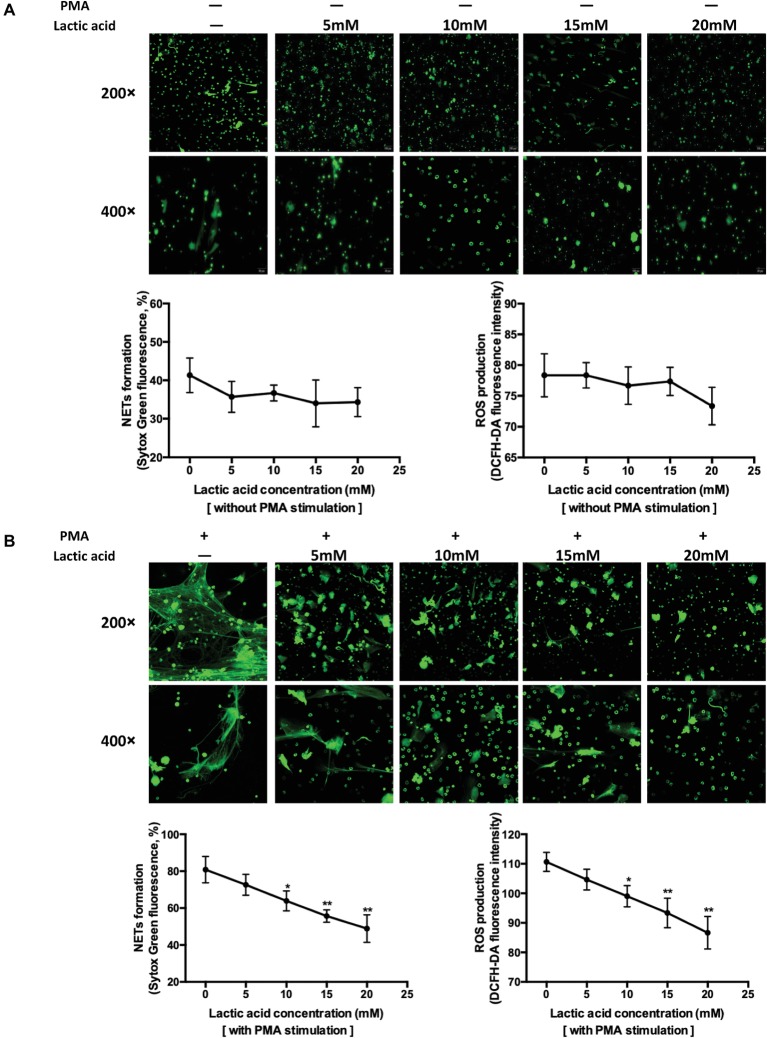
Increasing levels of lactic acid attenuated neutrophil extracellular traps release through inhibition of ROS production using *in vitro* methods. **(A)** Fresh neutrophils isolated from mouse bone marrow were seeded into 96-well plate and treated with media only, 5, 10, 15, and 20 mM lactic acid, respectively, for 4 h. The first row shows the structure of extracellular traps stained with SytoxGreen at a magnification of 200×. The second row is at a magnification of 400×. Extracellular DNA stained with SytoxGreen was hemi-quantified using a fluorescence microplate reader which indirectly represents NETs release. Intracellular ROS was detected with a cell probe and quantified using a fluorescence microplate reader at 480 nm excitation and 520 nm emission wavelength. The curve represents mean values (with SEM) with 100 nM PMA plus five different lactic acid concentrations (0, 5, 10, 15, 20 mM). * indicates *p* < 0.05, ** indicates *p* < 0.001, *** indicates *p* < 0.005, **** indicates *p* < 0.0001. **(B)** Neutrophils were seeded and treated with 100 nM PMA, 100 nM PMA plus 5, 10, 15, and 20 mM lactic acid, respectively, for 4 h. The curve represents mean values (with SEM) with 100 nM PMA plus five different lactic acid concentrations (0, 5, 10, 15, 20 mM). * indicates *p* < 0.05, ** indicates *p* < 0.001.

To improve interpretation of the data linked LA and NETs release at the cell level, neutrophils collected from the bone marrow were mixed with four LA concentrations (Figures 3A,B). PMA-stimulated neutrophils released the highest level of NETs, with levels decreasing as the LA concentration increased (PMA plus LA at 0, 5, 10, 15, and 20 mM, corresponding pH of media at 7.55, 6.86, 6.44, 6.0, and 5.51). ROS generation followed this same pattern. Two models of NETs formation are well known to date, suicidal NETs formation, and vital pathway with intact nuclear and plasma membrane, and these two are ROS dependent and ROS independent, respectively (Delgado-Rizo et al., 2017). LA impaired the NETs formation in our study was proved to be dependent on the diminishing effect of intracellular ROS production. Spontaneously, NETs formation *in vitro* is small and the decreasing trend of NETs formation with increasing LA concentration is relatively flat.

LA is a byproduct of anaerobic glycolysis and is typically 1–2 mM in plasma at rest. With intensive exercise, plasma LA can reach 20 mM (Abebayehu et al., 2016). LA promotes M2 macrophage polarization (Ohashi et al., 2017) and inhibits cytotoxic T cell function (Ohashi et al., 2013). The effects of LA on the capacity of neutrophils to release NETs have not been previously investigated. Extracellular acidification inhibits the ROS-dependent formation of NETs (Abebayehu et al., 2016). Extracellular acidosis (pH 6.5, 6.0, and 5.5) and intracellular acidification associated with diseased states inhibit the release of ROS-dependent NETs upon stimulation of neutrophils with PMA and immobilized immune complexes. These data are consistent with the findings described in this paper, but exercise-induced LA accumulation is physiologically different than the pathological state. The acidification of the internal environment caused by exercise is difficult to simulate well *in vitro* studies, as for the existence of blood buffer system. Considering this issue, better means of simulating the *in vivo* environment and cell metabolism analysis should be utilized in the future research.

Although NETs can effectively capture and kill pathogens, they are also double-edge swords as they can cause tissue damage and thus contribute to inflammation and pathogenesis (Nery and Tetelbom, 2016; Sorensen and Borregaard, 2016; Delgado-Rizo et al., 2017; Toussaint et al., 2017). Therefore, to maintain homeostasis, regulation of neutrophil effector mechanisms is essential in response to different external stimuli including strenuous exercise. NETosis is a multifactorial process, but detailed molecular mechanisms are not fully understood. In PMA-stimulated neutrophils, NOX2-dependent ROS have been shown to activate intracellular signaling cascades (Raf/MEK/ERK, Akt, and p38 MAPK), which mediates the release of NETs (Parker et al., 2012; Keshari et al., 2013; Douda et al., 2014; Behnen et al., 2017). Our results describe an important negative relationship between LA and NETs release, but the underlying mechanism and linkages to other immune responses remain to be elucidated.

Neutrophils protect the body from invasion of foreign microorganisms by releasing NETs. The impairment of this function after 145 min of exhaustive running may contribute to transient immune dysfunction and increased susceptibility to infections described as the “open window” period (Kakanis et al., 2010). Additional research will determine if the overtraining syndrome is associated with a chronic decrease in the capacity of neutrophils to release NETs.

## Ethics Statement

Animals were maintained and used in compliance with Ethics Committee for Animal Experimentation of Shanghai University of Sport in accordance with the Guide for the Care and Use of Laboratory Animals (Institute for Laboratory Animal Research, USA), and all experimental procedures were approved by the Ethics Committee of Shanghai University of Sport (approved number: 2018011).

## Author Contributions

PC, YC, YS, and HS contributed conception and design of the study. YS, QH, LY, HW, and TL performed the animals running protocol, *in vivo* experiments and cell culture researches. XZ, FL, DW, and YC conducted fluorescent image acquisition work. YS and DN performed the results analysis and the article composition.

### Conflict of Interest Statement

The authors declare that the research was conducted in the absence of any commercial or financial relationships that could be construed as a potential conflict of interest.

## Supplementary Material

Supplementary Figure S1 Percentages of Dead/Live cells and the purity of isolated neutrophils from mice bone marrow. PI-PE was used to distinguish the dead from live cells, the flow cytometry shows the live and dead cells account for 90 and 6.77%, respectively. CD11b-FITC and Ly6G-PE-cy7 fluorescent dye were used to mark the surface marker of neutrophils; the result shows the neutrophils of CD11b+Ly6G+ accounts for more than 85%.
