# Supplementary figures and images for: Lactic Acid Accumulation During Exhaustive Exercise Impairs Release of Neutrophil Extracellular Traps in Mice
Source: Front Physiol. 2019 Jun 12;10:709. doi: 10.3389/fphys.2019.00709 (PMC6585869; doi:10.3389/fphys.2019.00709)

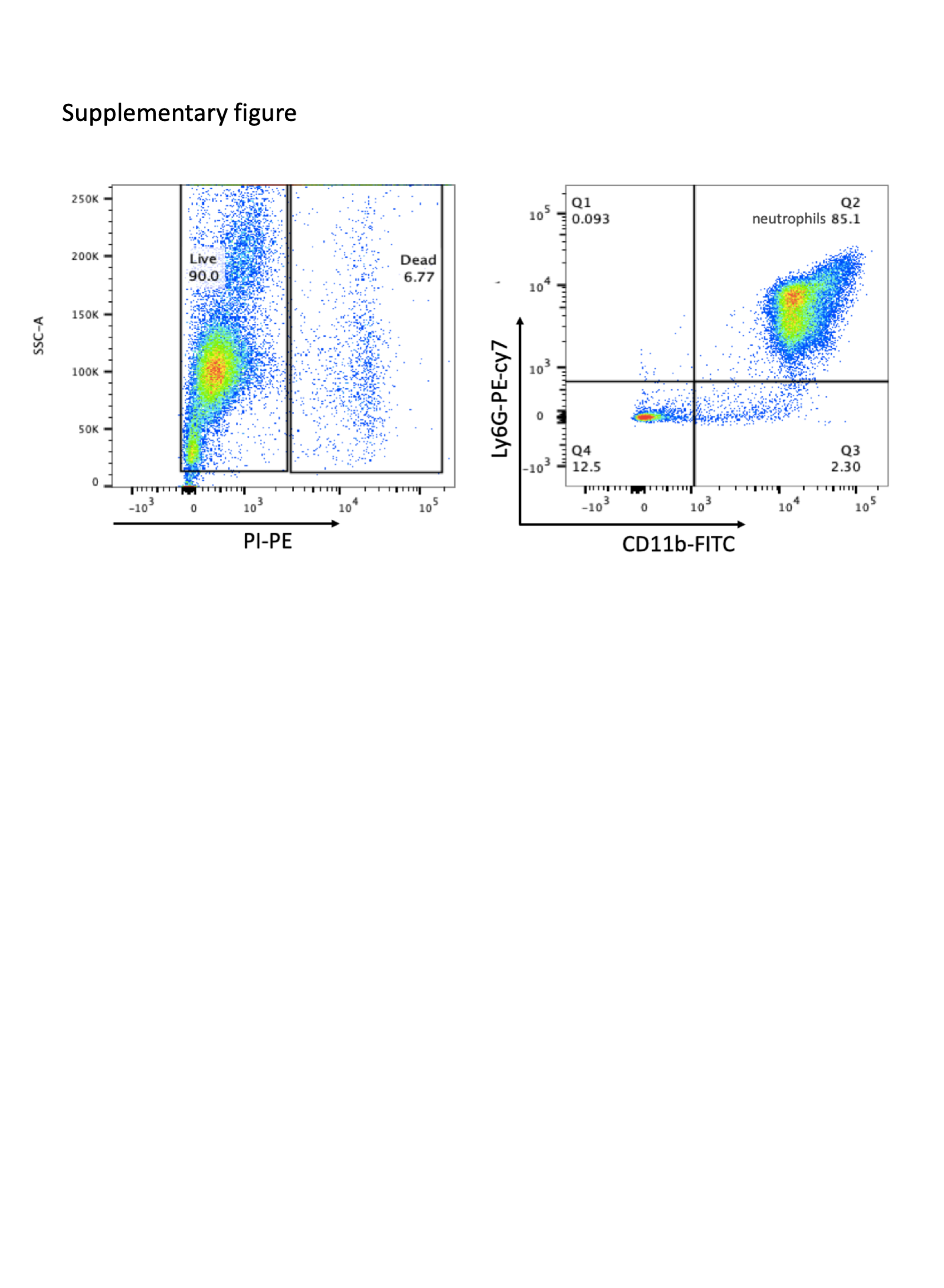

Supplement: Supplementary Figure S1 — Percentages of Dead/Live cells and the purity of isolated neutrophils from mice bone marrow. PI-PE was used to distinguish the dead from live cells, the flow cytometry shows the live and dead cells account for 90 and 6.77%, respectively. CD11b-FITC and Ly6G-PE-cy7 fluorescent dye were used to mark the surface marker of neutrophils; the result shows the neutrophils of CD11b+Ly6G+ accounts for more than 85%. [file Image_1.TIFF]
